# Structural elucidation and Hirshfeld surface analysis of a fused thio­phene ester: methyl 3-[(naphtho­[2,1-*b*]thio­phen-5-yl)meth­yl]-1-benzo­thio­phene-2-carboxyl­ate

**DOI:** 10.1107/S2056989026002239

**Published:** 2026-03-05

**Authors:** Sekaran Ranjith, Ayyamperumal Nataraj, Kabali Divya Bharathi, Arasambattu K. Mohanakrishnan

**Affiliations:** a Department of Physics SRM Institute of Science and Technology, Ramapuram Bharathi Salai Chennai 600089 Tamilnadu India; b Department of Organic Chemistry University of Madras, Guindy Campus Chennai - 600 025 Tamilnadu India; Vienna University of Technology, Austria

**Keywords:** crystal structure, benzo­thio­phene, inter­molecular inter­actions

## Abstract

The title compound, C_23_H_16_O_2_S_2_, is a benzo[*b*]thio­phene-2-carboxyl­ate derivative and consists of naphtho­thio­phene and benzo­thio­phene moieties bridged by a methyl­ene group. The dihedral angle between the two aromatic ring systems is 88.5 (2)°. Intra­molecular C—H⋯O inter­actions generate an *S*(6) ring motif. The acetate group assumes an extended conformation. Weak C—H⋯π and π–π stacking inter­actions are present in the crystal structure, together with a short S⋯S inter­action of 3.77 (8) Å. A Hirshfeld surface analysis indicates that H⋯H inter­actions contribute the most to the crystal packing (34.9%).Please give email addresses for all authors

## Abstract 2

In the title compound, the two aromatic ring systems are arranged almost perpendicular to each other [dihedral angle 88.5 (2)°].

## 1. Chemical context

Benzo­thio­phenes are important components of organic semiconductors (OSCs) due to their potential for elongated and highly delocalized electronic structures (Huang *et al.*, 2012 ▸). OSCs have consistently attracted attention for their distinctive properties, such as mechanical flexibility and chemical versatility (Katz *et al.*, 2001 ▸). In this regard, benzothieno[3,2-*b*]benzo­thio­phene derivatives are also highly promising materials for organic light-emitting diodes (OLEDs) (Izawa *et al.*, 2009 ▸) and organic field-effect transistors (OFET). In this context, we present here the synthesis, crystal structure and Hirshfeld surface analysis of the benzo­thio­phene derivative C_23_H_16_O_2_S_2_.
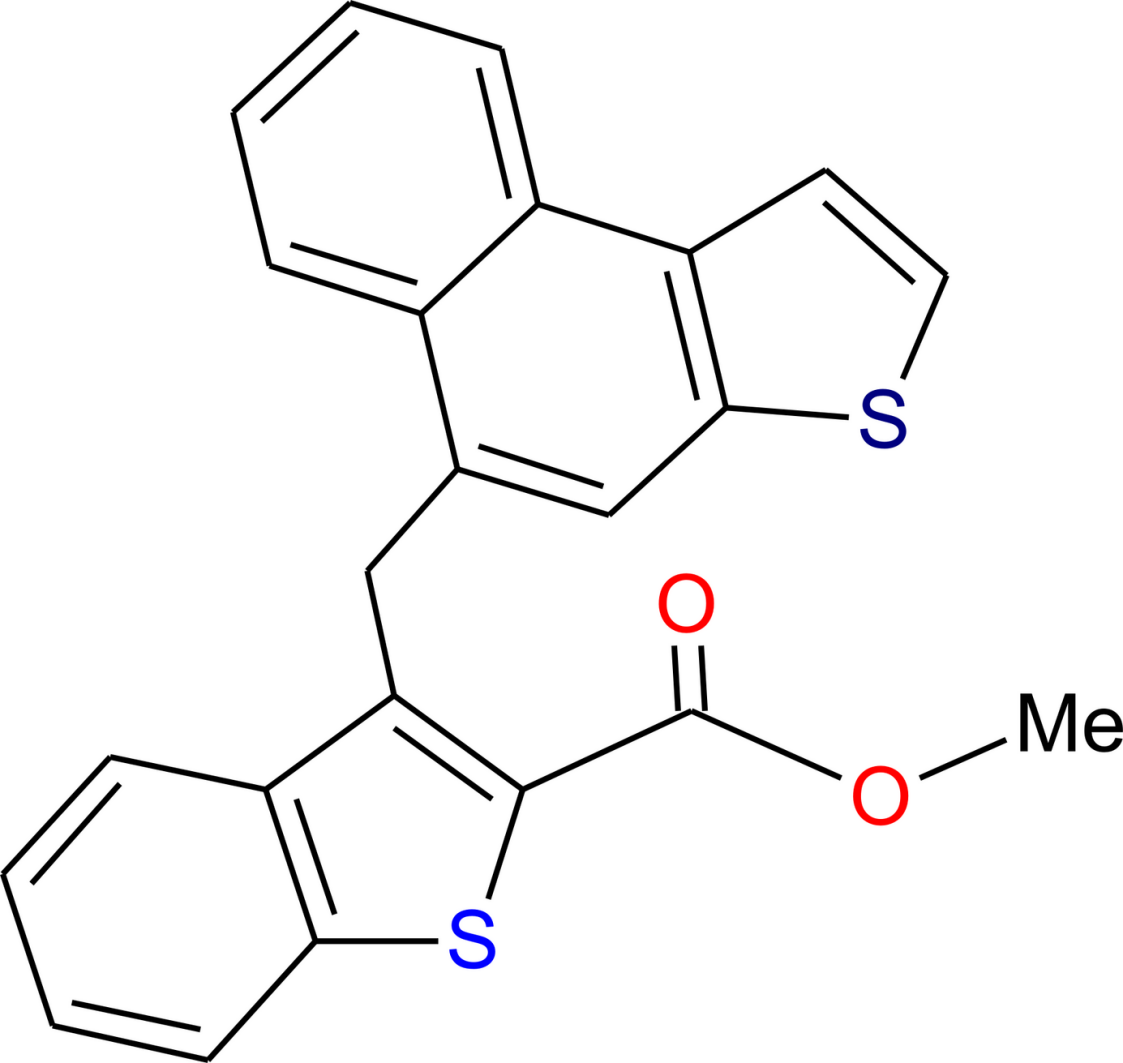


## 2. Structural commentary

**Figure 1:**
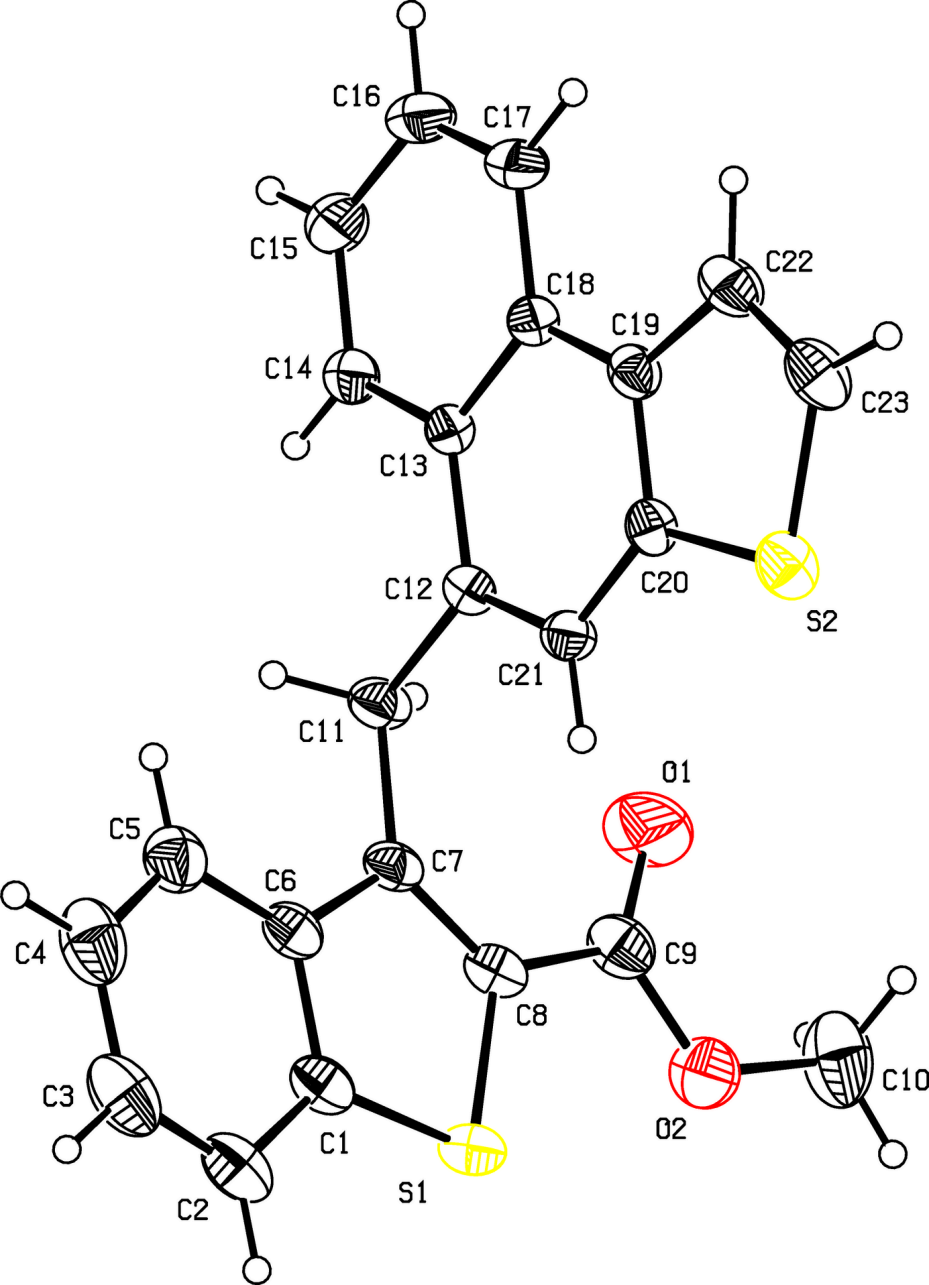
The mol­ecular structure of the title compound showing the atom-numbering scheme. Displacement ellipsoids are drawn at the 30% probability level.

**Figure 2:**
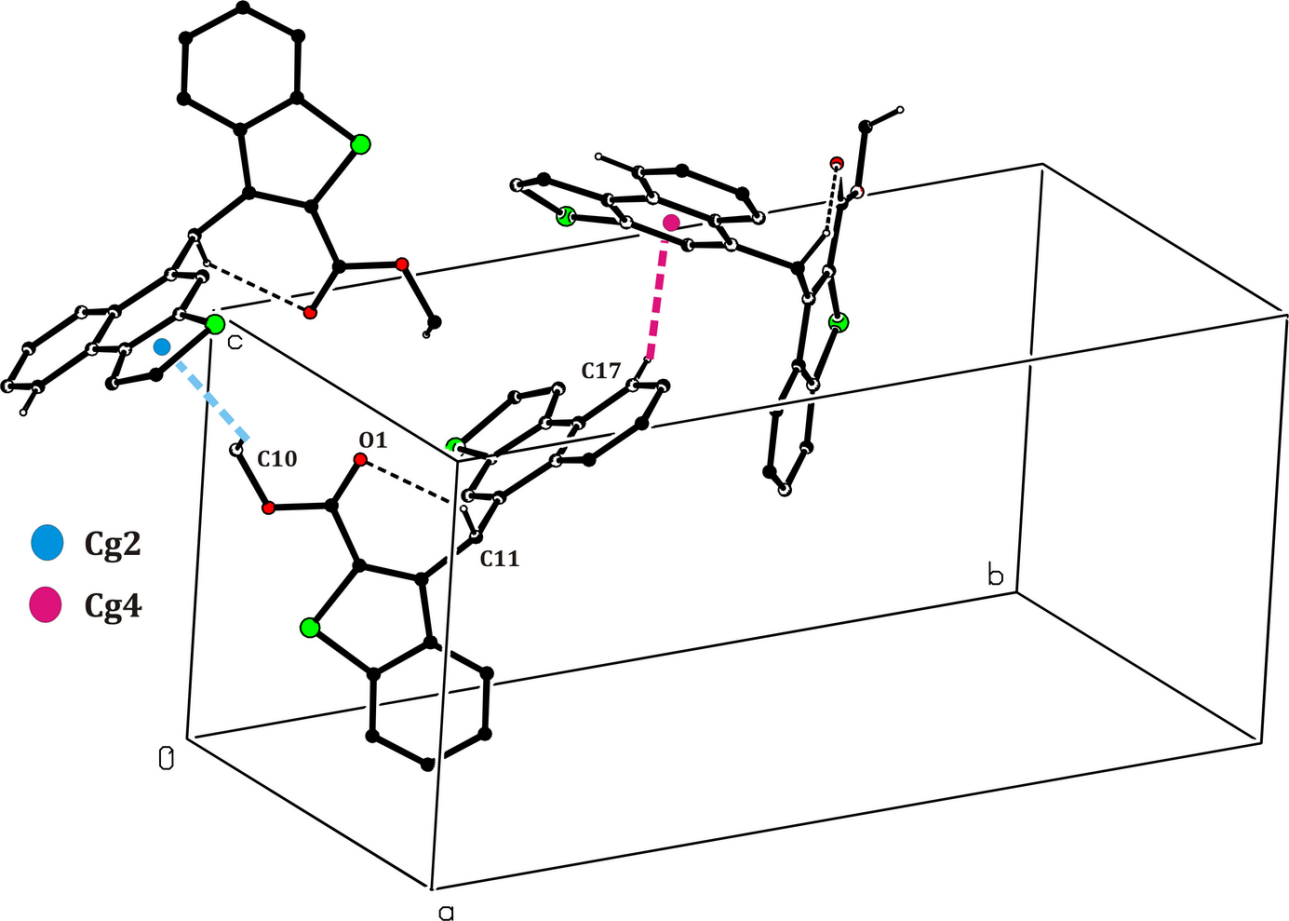
C—H⋯π and intra­molecular C—H⋯O inter­action (dashed line) in the title structure.

The mol­ecular structure of the title compound is displayed in Fig. 1 ▸. The naphtho­thio­phene group makes a dihedral angle of 88.5 (2)° with the benzo­thio­phene group. Atom S2 deviates by 0.064 (1) Å from the least-squares plane of the naphtho­thio­phene group; atom C8 deviates by −0.018 (2) Å from the least-squares plane of the benzo­thio­phene group, most probably due to the bulky substitution by an acetate moiety. The latter assumes an extended conformation as can be seen from the C10—O2—C9—C8 torsion angle of −175.2 (2)°. The benzo­thio­phene-2-carboxyl­ate moiety is nearly planar, with the largest deviation from the least-squares plane of 0.011 (2) Å for atom C7. The mol­ecular conformation is stabilized by a weak intra­molecular C11—H11*B*⋯O1hydrogen bond involving the methyl­ene group (C11) and the keto group (C9=O1) (Table 1 ▸, Fig. 2 ▸), which generates an *S*(6) ring motif (Bernstein *et al.*, 1995 ▸).

## 3. Supra­molecular features

**Figure 3:**
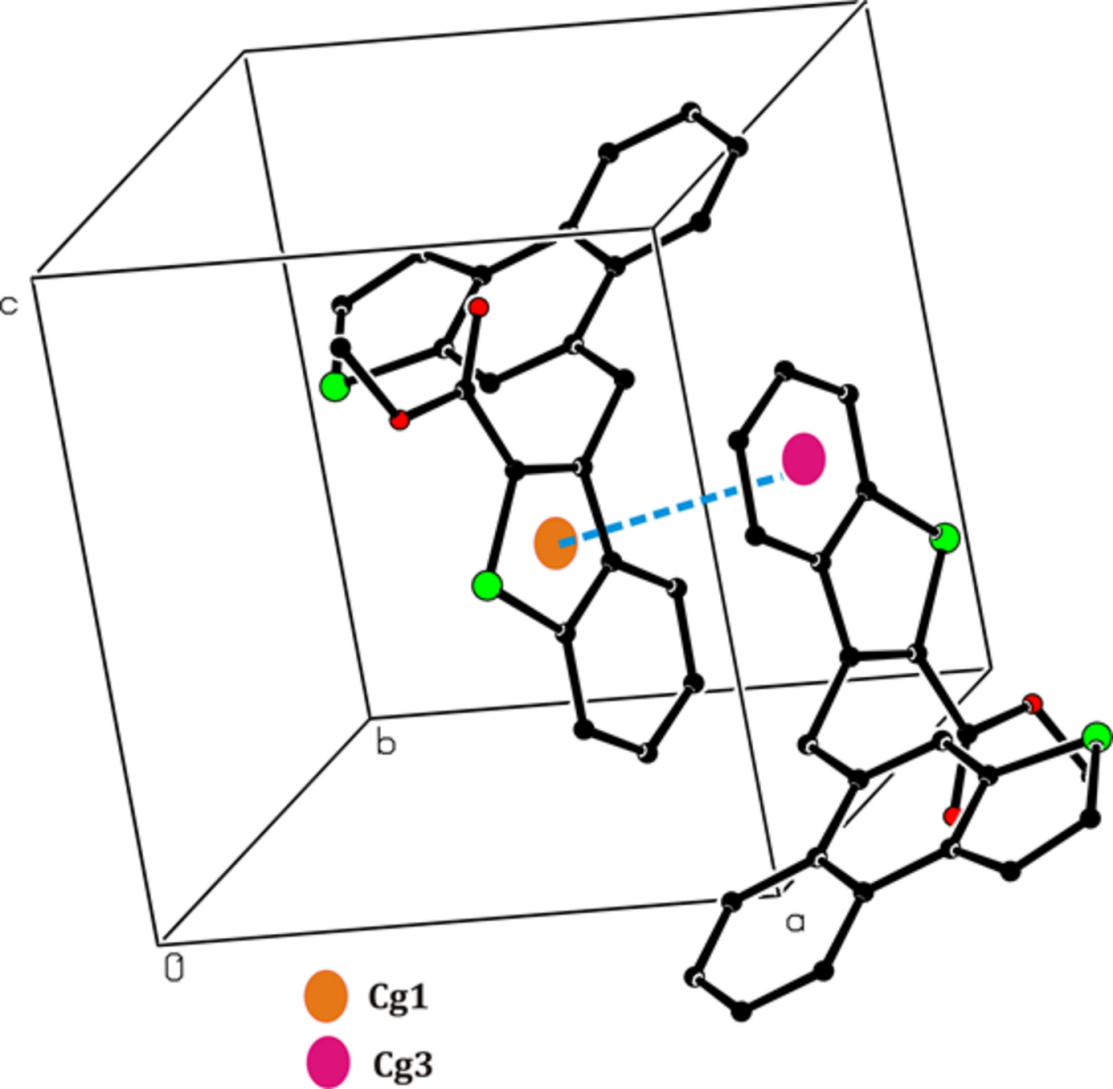
Relevant π–π inter­actions in the crystal of the title compound.

**Figure 4:**
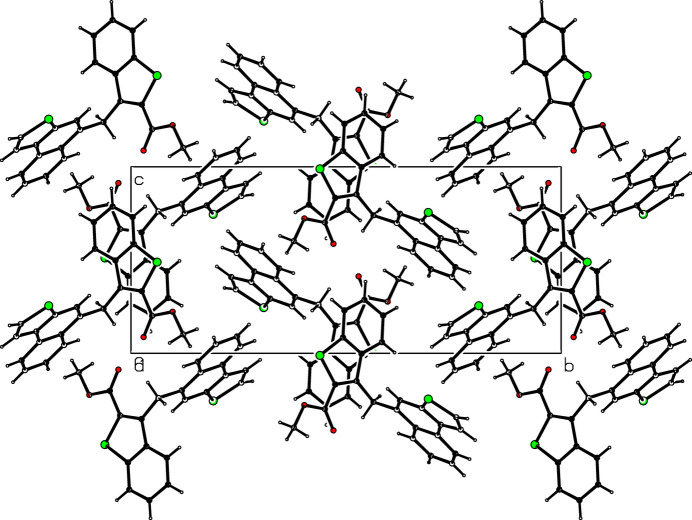
The mol­ecular packing viewed down the *a* axis.

There are no hydrogen-bonding inter­actions in the crystal. Instead, C—H⋯*Cg* inter­actions (*Cg* is the centre of gravity of an aromatic ring) are present, *viz*. C10—H10*A*⋯*Cg*2^i^ between the methyl group and the thio­phene ring (S2/C19/C20/C22/C23) attached to the naptho group, and C17—H17⋯*Cg*4^ii^ between a carbon atom of the naptho group and the central phenyl ring (C12/C13/C18-C21) of an adjacent naphtho­thio­phene group (Table 1 ▸, Fig. 2 ▸). In addition, π–π stacking is realized (Fig. 3 ▸) between the thio­phene ring of the benzo­thio­phene group (*Cg*1; S1,C1,C6–C8) and the phenyl ring (*Cg*3; C1–C6) of the benzo­thio­phene group of an adjacent mol­ecule (symmetry code: 2 − *x*, −*y*, 1 − *z*) at a *Cg*1⋯*Cg*3 distance of 3.9275 (2) Å with a slippage of 1.705 (3) Å. The mol­ecular packing is shown in Fig. 4 ▸.

## 4. Hirshfeld surface analysis

**Figure 5:**
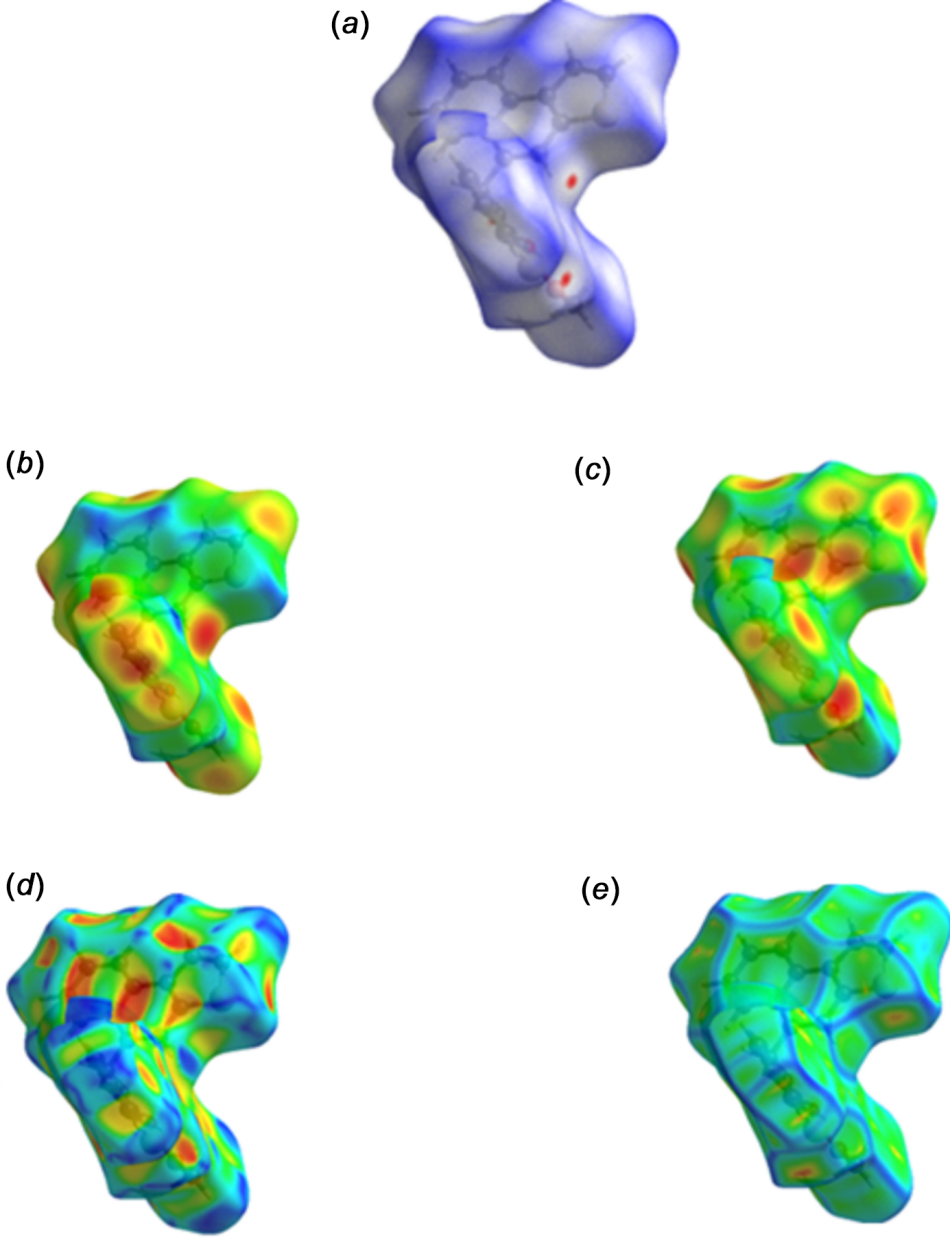
View of the three-dimensional Hirshfeld surface of the title compound mapped over (*a*) *d*_norm_, (*b*) *d*_i_, (*c*) *d*_e_, (*d*) shape index and (*e*) curvedness.

**Figure 6:**
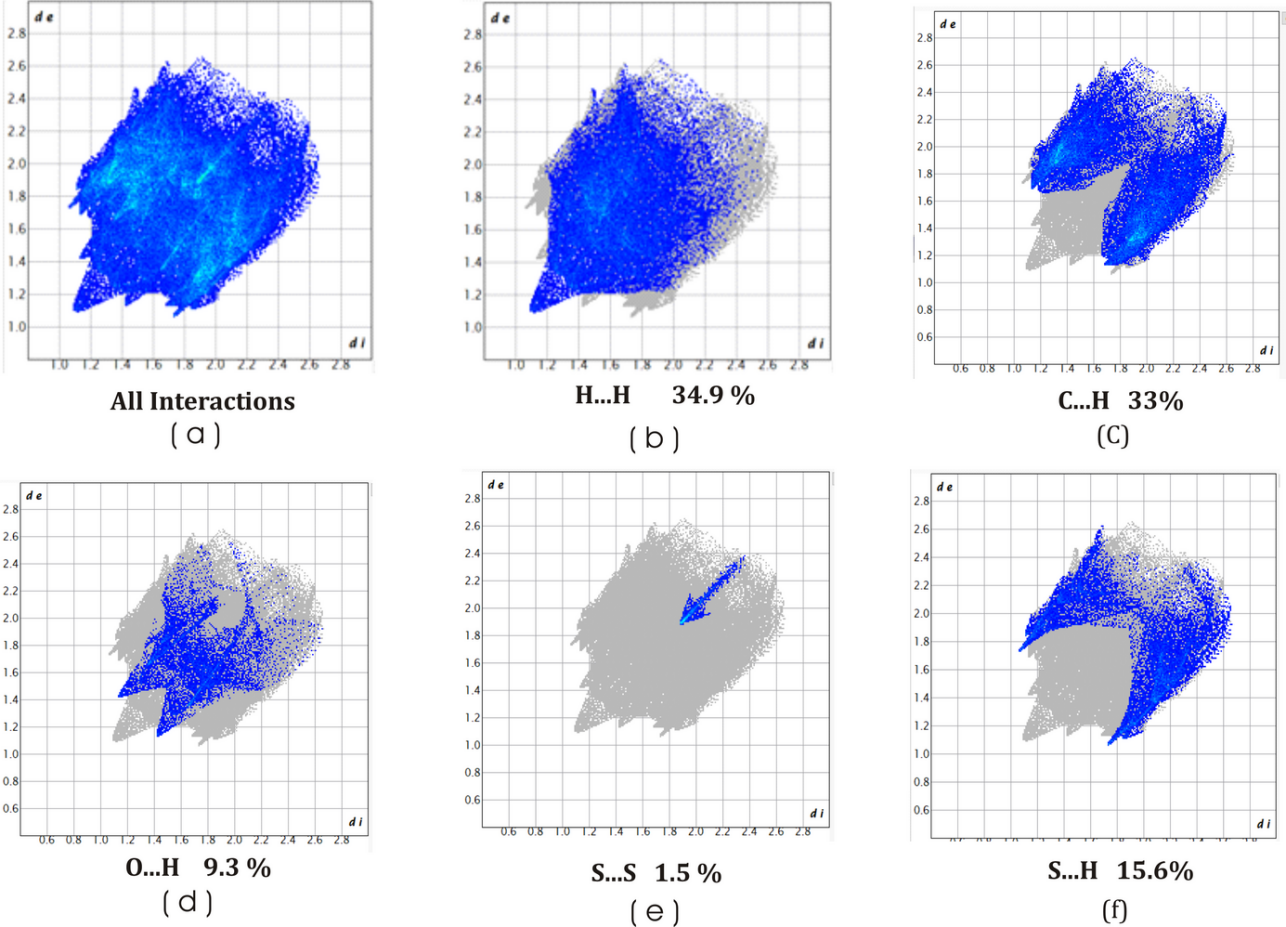
Two-dimensional fingerprint plots for the compound, showing (*a*) all inter­actions, and delineated into (*b*) H⋯H (*c*) C⋯H (*d*) H⋯O / O⋯H, (*e*) S⋯S and (*f*) S⋯H inter­actions. The *d*_i_ and *d*_e_ values are the closest inter­nal and external distances (in Å) from given points on the Hirshfeld surface.

Hirshfeld surface (HS) analysis (Hirshfeld, 1977 ▸) was carried out using *CrystalExplorer* (Spackman *et al.*, 2021 ▸). In the HS plotted over *d*_norm_, the white surface indicates contacts with distances equal to the sum of the van der Waals radii, and the red and blue colours indicate distances shorter (in close contact) or longer (distinct contacts) than the van der Waals radii, respectively (Venkatesan *et al.*, 2016 ▸). The Hirshfeld surfaces plotted over different qu­anti­ties are depicted in Fig. 5 ▸. Two-dimensional fingerprint plots showing the occurrence of all inter­molecular contacts (McKinnon *et al.*, 2007 ▸) are presented in Fig. 6 ▸. The most important inter­action originates from H⋯H contacts, contributing 34.9% to the overall crystal packing, which is reflected as widely scattered points of high density due to the large hydrogen content of the mol­ecule. Almost as significant is the contribution from the C⋯H/H⋯C inter­actions (33.0%), indicating that the C—H⋯π inter­actions contribute significantly, likely hydrogen atoms inter­act with the π-electron-rich region to favour layered or offset stacking. Through electrostatic stabilization assisted by dispersion, three-dimensional packing efficiency is enhanced with a slight directionality. More contributions due to S⋯H/H⋯S inter­actions (15.6%) stem from the polarizability of the sulfur atom in weak hydrogen bond-like contacts. Sulfur atoms have soft donor properties, which allow them to participate in stabilizing inter­molecular inter­actions. In the same way, O⋯H/H⋯O inter­actions (9.3%) define classical weak hydrogen-bonding pathways mediated by electronegative oxygen atoms. Electrostatic stabilization occurs as a consequence of these contacts and arrangement of mol­ecules in the lattice in a suitable manner. This lessens the rotational and translational degrees of freedom. The S⋯S contacts (1.5%) represent only a small percentage, but they are structurally important. The presence of short chalcogen–chalcogen contacts is known to enhance the lattice compactness via favourable overlap of their orbitals and associated dispersion inter­actions that lead to local densification. Such changes also promote packing rigidity. These inter­actions can often serve as reinforcing elements of the packing within a crystal. When combined, these inter­actions give rise to a hierarchy of stabilization. The global stabilization is induced by the H⋯H dispersion forces, while the mol­ecular stacking is being controlled by C—H⋯π inter­actions. Also, the directional locking and local reinforcement is conferred by the heteroatom-mediated contacts (S⋯H, O⋯H, S⋯S).

**Figure 7:**
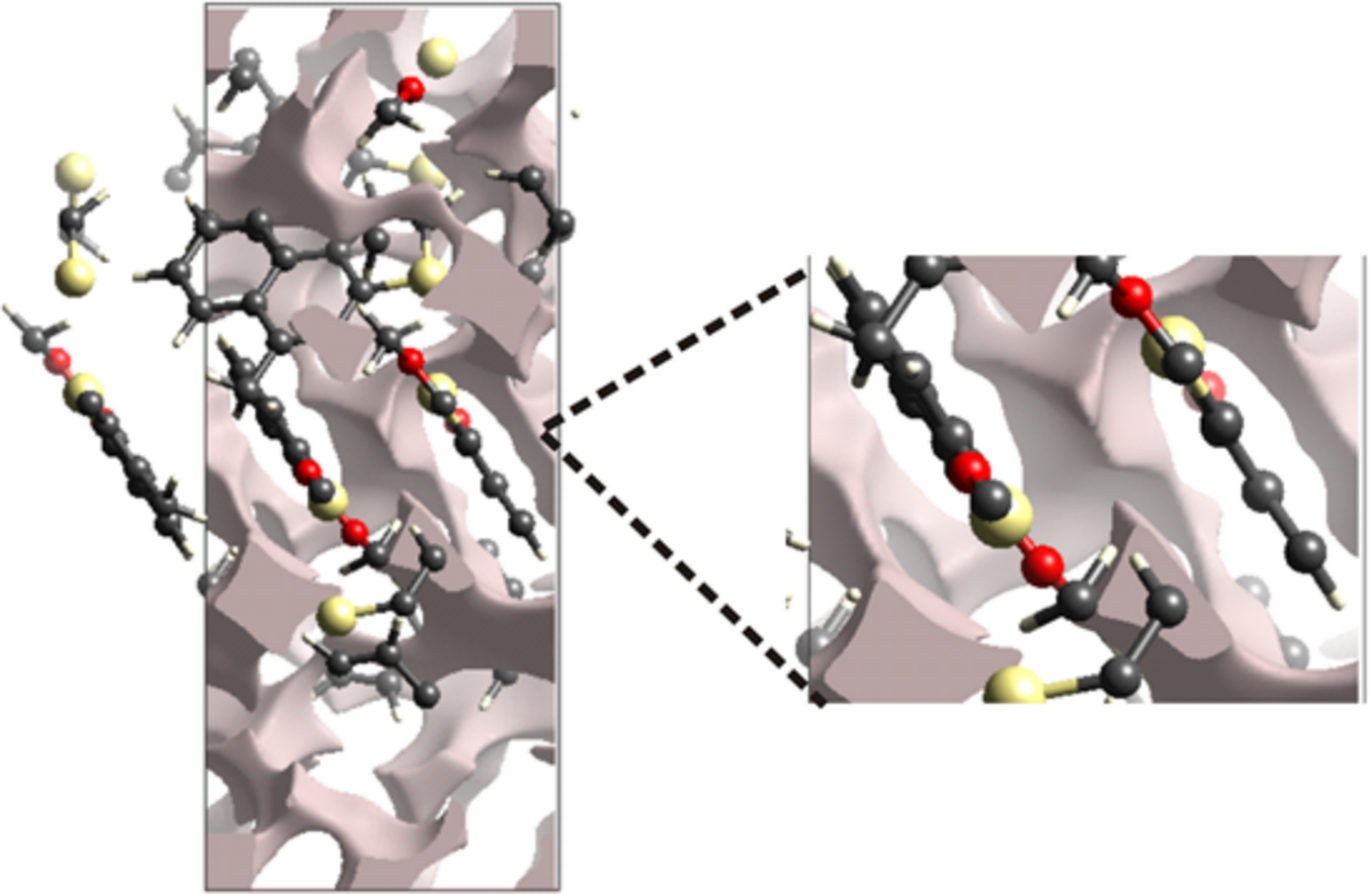
Plot showing the crystal voids in the crystal structure.

To analyse the crystal mechanical stability of the title crystal, a void evaluation was performed by summing the electron densities of the spherically symmetric atoms included in the asymmetric unit (Fig. 7 ▸). The void surface is recognized as an isosurface. The crystal voids within the unit cell are characterized by their volume and surface, which are 213.21 Å^3^ and 654.88 Å^2^, respectively. Considering the crystal volume of 1861.12 Å^3^, the proportion of void space within the unit cell is 11.45%.

## 5. Database survey

A search of the Cambridge Structural Database (Version 5.37; Groom *et al.*, 2016 ▸). for benzo­thio­phene-2-carboxyl­ate moieties resulted in 18 hits. Entries CUDLEV (Ivachtchenko *et al.*, 2019 ▸) and QAVXOD (Shen *et al.*, 2017 ▸) are the closest analogues of the title compound. CUDLEV crystallizes in the monoclinic space group *P*2_1_/*c*. QAVXOD crystallizes in the monoclinic space group *P*2_1_/*n*. In CUDLEV, all atoms of the benzo­thio­phene fragment lie in the plane within 0.02 Å. In CUDLEV, the ester substituent is turned significantly to the bicyclic system and the mol­ecules are bound by very weak C—H⋯O inter­molecular hydrogen bonds. Both related structures are distinguished by the nature of their substituents (morpholine-4-sulfonyl and biphenyl-4-yl groups, respectively), thus reflecting the structural flexibility of these compounds.

## 6. Synthesis and crystallization

The domino reaction of methyl 3-(bromo­meth­yl)-1-benzo­thio­phene-2-carboxyl­ate (0.3 g, 1.05 mmol) with naphtho­[2,1-*b*]thio­phene (0.19 g, 1.05 mmol) using ZnBr_2_ (0.47 g, 2.10 mmol) was carried out in dry 1,2-di­chloro­ethane (10 ml) at room temperature under N_2_ atmosphere for 6 h. The reaction mixture was then poured into crushed ice (50 g) and acidified with conc. HCl (2 ml). The crude product was then extracted with di­chloro­methane (3 × 10 ml) and dried with Na_2_SO_4_. The subsequent removal of the solvent *in vacuo* was followed by column chromatographic purification on silica gel (eluent: 2% ethyl acetate in hexa­ne) afforded the title compound (0.23 g, 69%) as a colorless solid. Single crystals suitable for X-ray diffraction were obtained by slow evaporation of a solution in ethyl acetate at room temperature.

## 7. Refinement

Crystal data, data collection and structure refinement details are summarized in Table 2 ▸. All hydrogen atoms were positioned geometrically and refined as riding with C—H = 0.93 Å (aromatic CH) with *U*_iso_(H) = 1.5*U*_eq_(C) for methyl groups and 1.2*U*_eq_(C) for other H atoms.

## Supplementary Material

Crystal structure: contains datablock(s) shelx, I. DOI: 10.1107/S2056989026002239/wm5787sup1.cif

Structure factors: contains datablock(s) I. DOI: 10.1107/S2056989026002239/wm5787Isup2.hkl

Supporting information file. DOI: 10.1107/S2056989026002239/wm5787Isup3.cml

CCDC reference: 2479318

Additional supporting information:  crystallographic information; 3D view; checkCIF report

## Figures and Tables

**Table 1:** Hydrogen-bond geometry (Å, °) *Cg*2 and *Cg*4 are the centroids of the S2/C19/C20/C22/C23 and C12/C13/C18-C21 rings, respectively.

*D*—H⋯*A*	*D*—H	H⋯*A*	*D*⋯*A*	*D*—H⋯*A*
C11—H11*B*⋯O1	0.97	2.44	3.007 (3)	117
C10—H10*A*⋯*Cg*2^i^	0.96	2.86	3.397 (3)	116
C17—H17⋯*Cg*4^ii^	0.93	2.98	3.689 (2)	134

Symmetry codes: (i) 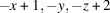; (ii) 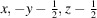.

**Table 2:** Experimental details

Crystal data	
Chemical formula	C_23_H_16_O_2_S_2_
*M*_r_	388.48
Crystal system, space group	Monoclinic, *P*2_1_/*c*
Temperature (K)	293
*a*, *b*, *c* (Å)	8.8381 (4), 22.0387 (9), 9.5910 (3)
β (°)	94.968 (1)
*V* (Å^3^)	1861.12 (13)
*Z*	4
Radiation type	Mo *K*α
μ (mm^−1^)	0.30
Crystal size (mm)	0.27 × 0.12 × 0.08

Data collection	
Diffractometer	Bruker APEXII CCD area detector diffractometer
Absorption correction	Multi-scan (*SADABS*; Krause *et al.*, 2015 ▸)
*T*_min_, *T*_max_	0.922, 0.976
No. of measured, independent and observed [*I* > 2σ(*I*)] reflections	38099, 4271, 3717
*R*_int_	0.068
(sin θ/λ)_max_ (Å^−1^)	0.651

Refinement	
*R*[*F*^2^ > 2σ(*F*^2^)], *wR*(*F*^2^), *S*	0.047, 0.122, 1.06
No. of reflections	4271
No. of parameters	246
H-atom treatment	H-atom parameters constrained
Δρ_max_, Δρ_min_ (e Å^−3^)	0.42, −0.30

Computer programs: *APEX4* and *SAINT* (Bruker, 2018 ▸), *SHELXS* (Sheldrick, 2008 ▸), *SHELXL* (Sheldrick, 2015 ▸), *ORTEP-3 for Windows* (Farrugia, 2012 ▸) and *PLATON* (Spek, 2020 ▸).

## supplementary crystallographic information

### Methyl 3-[(naphtho[2,1-b]thiophen-5-yl)methyl]-1-benzothiophene-2-carboxylate Crystal data

**Table:**

C_23_H_16_O_2_S_2_	*F*(000) = 808
*M**_r_* = 388.48	*D*_x_ = 1.386 Mg m^−^^3^
Monoclinic, *P*2_1_/*c*	Mo *K*α radiation, λ = 0.71073 Å
*a* = 8.8381 (4) Å	Cell parameters from 3717 reflections
*b* = 22.0387 (9) Å	θ = 2.5–27.6°
*c* = 9.5910 (3) Å	µ = 0.30 mm^−^^1^
β = 94.968 (1)°	*T* = 293 K
*V* = 1861.12 (13) Å^3^	Block, colourless
*Z* = 4	0.27 × 0.12 × 0.08 mm

### Methyl 3-[(naphtho[2,1-b]thiophen-5-yl)methyl]-1-benzothiophene-2-carboxylate Data collection

**Table:**

Bruker APEXII CCD area detector diffractometer	3717 reflections with *I* > 2σ(*I*)
ω and φ scans	*R*_int_ = 0.068
Absorption correction: multi-scan (SADABS; Krause *et al.*, 2015)	θ_max_ = 27.6°, θ_min_ = 2.5°
*T*_min_ = 0.922, *T*_max_ = 0.976	*h* = −11→11
38099 measured reflections	*k* = −28→28
4271 independent reflections	*l* = −12→12

### Methyl 3-[(naphtho[2,1-b]thiophen-5-yl)methyl]-1-benzothiophene-2-carboxylate Refinement

**Table:**

Refinement on *F*^2^	0 restraints
Least-squares matrix: full	Hydrogen site location: inferred from neighbouring sites
*R*[*F*^2^ > 2σ(*F*^2^)] = 0.047	H-atom parameters constrained
*wR*(*F*^2^) = 0.122	*w* = 1/[σ^2^(*F*_o_^2^) + (0.0483*P*)^2^ + 0.840*P*] where *P* = (*F*_o_^2^ + 2*F*_c_^2^)/3
*S* = 1.06	(Δ/σ)_max_ < 0.001
4271 reflections	Δρ_max_ = 0.42 e Å^−^^3^
246 parameters	Δρ_min_ = −0.30 e Å^−^^3^

### Methyl 3-[(naphtho[2,1-b]thiophen-5-yl)methyl]-1-benzothiophene-2-carboxylate Special details

**Table:**

Geometry. All esds (except the esd in the dihedral angle between two l.s. planes) are estimated using the full covariance matrix. The cell esds are taken into account individually in the estimation of esds in distances, angles and torsion angles; correlations between esds in cell parameters are only used when they are defined by crystal symmetry. An approximate (isotropic) treatment of cell esds is used for estimating esds involving l.s. planes.

### Methyl 3-[(naphtho[2,1-b]thiophen-5-yl)methyl]-1-benzothiophene-2-carboxylate Fractional atomic coordinates and isotropic or equivalent isotropic displacement parameters (Å^2^)

**Table:**

	*x*	*y*	*z*	*U*_iso_*/*U*_eq_	
S1	0.34542 (6)	0.43934 (2)	0.98904 (5)	0.05024 (18)	
S2	0.62927 (6)	0.69125 (3)	0.75510 (5)	0.05271 (18)	
C12	0.20885 (19)	0.61820 (7)	0.69839 (17)	0.0356 (4)	
C18	0.2214 (2)	0.71310 (8)	0.56103 (18)	0.0388 (4)	
C21	0.35626 (19)	0.62550 (8)	0.74821 (18)	0.0390 (4)	
H21	0.402611	0.597043	0.809487	0.047*	
C13	0.13745 (19)	0.66204 (7)	0.60185 (17)	0.0357 (4)	
C20	0.43935 (19)	0.67627 (8)	0.70710 (17)	0.0377 (4)	
C11	0.1169 (2)	0.56398 (9)	0.7392 (2)	0.0463 (4)	
H11A	0.021974	0.578523	0.770724	0.056*	
H11B	0.092280	0.539324	0.656601	0.056*	
C19	0.3754 (2)	0.72038 (8)	0.61743 (18)	0.0392 (4)	
C8	0.2809 (2)	0.47487 (8)	0.83337 (19)	0.0449 (4)	
C7	0.1938 (2)	0.52451 (8)	0.85167 (19)	0.0407 (4)	
C6	0.1804 (2)	0.53600 (8)	0.9982 (2)	0.0451 (4)	
O2	0.4202 (2)	0.40316 (7)	0.72290 (17)	0.0672 (4)	
C14	−0.0138 (2)	0.65562 (9)	0.5444 (2)	0.0460 (4)	
H14	−0.070818	0.622626	0.570122	0.055*	
C22	0.4825 (3)	0.76670 (9)	0.5912 (2)	0.0534 (5)	
H22	0.459031	0.800284	0.534715	0.064*	
C17	0.1512 (2)	0.75430 (10)	0.4623 (2)	0.0559 (5)	
H17	0.206170	0.787242	0.433174	0.067*	
C1	0.2574 (2)	0.49295 (9)	1.0853 (2)	0.0484 (5)	
C15	−0.0784 (2)	0.69733 (11)	0.4510 (2)	0.0597 (6)	
H15	−0.178874	0.692640	0.415114	0.072*	
C9	0.3271 (3)	0.45074 (10)	0.7011 (2)	0.0550 (5)	
C23	0.6207 (3)	0.75655 (10)	0.6568 (2)	0.0577 (5)	
H23	0.703206	0.782216	0.649718	0.069*	
C5	0.1042 (3)	0.58349 (10)	1.0601 (2)	0.0576 (5)	
H5	0.052588	0.612757	1.004688	0.069*	
C3	0.1833 (3)	0.54270 (13)	1.2871 (3)	0.0724 (7)	
H3	0.183439	0.545460	1.383885	0.087*	
C2	0.2580 (3)	0.49621 (11)	1.2304 (2)	0.0604 (6)	
H2	0.308492	0.467139	1.287243	0.072*	
O1	0.2888 (3)	0.47072 (10)	0.58721 (17)	0.0950 (7)	
C4	0.1066 (3)	0.58634 (13)	1.2031 (3)	0.0750 (7)	
H4	0.056388	0.617783	1.244485	0.090*	
C16	0.0055 (3)	0.74655 (11)	0.4097 (3)	0.0663 (6)	
H16	−0.038849	0.774302	0.345523	0.080*	
C10	0.4826 (4)	0.37858 (14)	0.6016 (3)	0.0848 (9)	
H10A	0.402080	0.362650	0.538502	0.127*	
H10B	0.552652	0.346662	0.629634	0.127*	
H10C	0.534757	0.410000	0.555647	0.127*	

### Methyl 3-[(naphtho[2,1-b]thiophen-5-yl)methyl]-1-benzothiophene-2-carboxylate Atomic displacement parameters (Å^2^)

**Table:**

	*U*^11^	*U*^22^	*U*^33^	*U*^12^	*U*^13^	*U*^23^
S1	0.0610 (3)	0.0447 (3)	0.0441 (3)	0.0015 (2)	−0.0002 (2)	0.01029 (19)
S2	0.0415 (3)	0.0644 (3)	0.0513 (3)	−0.0154 (2)	−0.0015 (2)	0.0013 (2)
C12	0.0382 (8)	0.0342 (8)	0.0347 (8)	−0.0025 (7)	0.0046 (6)	0.0031 (6)
C18	0.0417 (9)	0.0341 (8)	0.0417 (9)	0.0039 (7)	0.0095 (7)	0.0038 (7)
C21	0.0385 (9)	0.0397 (9)	0.0385 (8)	−0.0011 (7)	0.0011 (7)	0.0060 (7)
C13	0.0373 (8)	0.0355 (8)	0.0350 (8)	0.0023 (6)	0.0071 (6)	0.0021 (6)
C20	0.0360 (8)	0.0417 (9)	0.0357 (8)	−0.0051 (7)	0.0039 (6)	−0.0027 (7)
C11	0.0429 (10)	0.0451 (10)	0.0497 (10)	−0.0087 (8)	−0.0017 (8)	0.0133 (8)
C19	0.0444 (9)	0.0346 (8)	0.0397 (9)	−0.0036 (7)	0.0098 (7)	−0.0013 (7)
C8	0.0537 (11)	0.0416 (9)	0.0389 (9)	−0.0077 (8)	0.0022 (8)	0.0064 (7)
C7	0.0428 (9)	0.0348 (9)	0.0440 (9)	−0.0096 (7)	0.0020 (7)	0.0084 (7)
C6	0.0476 (10)	0.0403 (9)	0.0479 (10)	−0.0133 (8)	0.0064 (8)	0.0041 (8)
O2	0.0871 (12)	0.0582 (9)	0.0584 (9)	0.0076 (8)	0.0173 (8)	−0.0084 (7)
C14	0.0388 (9)	0.0500 (10)	0.0494 (10)	0.0007 (8)	0.0045 (8)	0.0096 (8)
C22	0.0599 (12)	0.0437 (10)	0.0576 (12)	−0.0141 (9)	0.0114 (9)	0.0052 (9)
C17	0.0540 (12)	0.0453 (11)	0.0691 (13)	0.0046 (9)	0.0102 (10)	0.0228 (10)
C1	0.0546 (11)	0.0476 (10)	0.0431 (10)	−0.0133 (9)	0.0040 (8)	0.0044 (8)
C15	0.0395 (10)	0.0728 (14)	0.0656 (13)	0.0062 (10)	−0.0018 (9)	0.0188 (11)
C9	0.0650 (13)	0.0511 (12)	0.0492 (11)	−0.0099 (10)	0.0063 (9)	−0.0003 (9)
C23	0.0567 (12)	0.0579 (12)	0.0593 (12)	−0.0260 (10)	0.0092 (10)	−0.0006 (10)
C5	0.0621 (13)	0.0470 (11)	0.0655 (13)	−0.0057 (10)	0.0162 (10)	−0.0015 (9)
C3	0.0880 (18)	0.0823 (17)	0.0487 (12)	−0.0267 (15)	0.0168 (12)	−0.0091 (12)
C2	0.0714 (14)	0.0693 (14)	0.0408 (10)	−0.0208 (11)	0.0061 (9)	0.0033 (10)
O1	0.1355 (19)	0.1086 (16)	0.0410 (9)	0.0244 (14)	0.0078 (10)	0.0073 (9)
C4	0.0850 (18)	0.0702 (16)	0.0741 (16)	−0.0156 (14)	0.0316 (14)	−0.0211 (13)
C16	0.0545 (13)	0.0663 (14)	0.0773 (15)	0.0120 (11)	0.0016 (11)	0.0347 (12)
C10	0.092 (2)	0.0868 (19)	0.0798 (18)	−0.0116 (16)	0.0312 (15)	−0.0345 (15)

### Methyl 3-[(naphtho[2,1-b]thiophen-5-yl)methyl]-1-benzothiophene-2-carboxylate Geometric parameters (Å, º)

**Table:**

S1—C1	1.726 (2)	O2—C10	1.436 (3)
S1—C8	1.7377 (18)	C14—C15	1.373 (3)
S2—C23	1.719 (2)	C14—H14	0.9300
S2—C20	1.7334 (17)	C22—C23	1.343 (3)
C12—C21	1.358 (2)	C22—H22	0.9300
C12—C13	1.445 (2)	C17—C16	1.353 (3)
C12—C11	1.516 (2)	C17—H17	0.9300
C18—C17	1.416 (3)	C1—C2	1.393 (3)
C18—C13	1.421 (2)	C15—C16	1.391 (3)
C18—C19	1.430 (3)	C15—H15	0.9300
C21—C20	1.413 (2)	C9—O1	1.199 (3)
C21—H21	0.9300	C23—H23	0.9300
C13—C14	1.408 (2)	C5—C4	1.371 (4)
C20—C19	1.385 (2)	C5—H5	0.9300
C11—C7	1.502 (2)	C3—C2	1.358 (4)
C11—H11A	0.9700	C3—C4	1.393 (4)
C11—H11B	0.9700	C3—H3	0.9300
C19—C22	1.429 (2)	C2—H2	0.9300
C8—C7	1.358 (3)	C4—H4	0.9300
C8—C9	1.466 (3)	C16—H16	0.9300
C7—C6	1.443 (3)	C10—H10A	0.9600
C6—C1	1.401 (3)	C10—H10B	0.9600
C6—C5	1.404 (3)	C10—H10C	0.9600
O2—C9	1.338 (3)		
			
C1—S1—C8	91.25 (9)	C23—C22—C19	112.82 (19)
C23—S2—C20	91.03 (10)	C23—C22—H22	123.6
C21—C12—C13	119.91 (15)	C19—C22—H22	123.6
C21—C12—C11	121.49 (15)	C16—C17—C18	121.10 (19)
C13—C12—C11	118.58 (15)	C16—C17—H17	119.4
C17—C18—C13	118.88 (17)	C18—C17—H17	119.4
C17—C18—C19	121.93 (17)	C2—C1—C6	121.4 (2)
C13—C18—C19	119.16 (15)	C2—C1—S1	127.37 (18)
C12—C21—C20	120.11 (16)	C6—C1—S1	111.26 (14)
C12—C21—H21	119.9	C14—C15—C16	120.4 (2)
C20—C21—H21	119.9	C14—C15—H15	119.8
C14—C13—C18	118.18 (16)	C16—C15—H15	119.8
C14—C13—C12	122.05 (16)	O1—C9—O2	123.5 (2)
C18—C13—C12	119.76 (15)	O1—C9—C8	125.3 (2)
C19—C20—C21	122.34 (16)	O2—C9—C8	111.24 (18)
C19—C20—S2	111.39 (13)	C22—C23—S2	113.05 (15)
C21—C20—S2	126.25 (14)	C22—C23—H23	123.5
C7—C11—C12	115.01 (15)	S2—C23—H23	123.5
C7—C11—H11A	108.5	C4—C5—C6	119.4 (2)
C12—C11—H11A	108.5	C4—C5—H5	120.3
C7—C11—H11B	108.5	C6—C5—H5	120.3
C12—C11—H11B	108.5	C2—C3—C4	121.2 (2)
H11A—C11—H11B	107.5	C2—C3—H3	119.4
C20—C19—C22	111.70 (17)	C4—C3—H3	119.4
C20—C19—C18	118.67 (15)	C3—C2—C1	118.6 (2)
C22—C19—C18	129.57 (17)	C3—C2—H2	120.7
C7—C8—C9	127.41 (18)	C1—C2—H2	120.7
C7—C8—S1	113.54 (14)	C5—C4—C3	120.8 (2)
C9—C8—S1	119.02 (15)	C5—C4—H4	119.6
C8—C7—C6	111.25 (16)	C3—C4—H4	119.6
C8—C7—C11	126.90 (17)	C17—C16—C15	120.40 (19)
C6—C7—C11	121.84 (17)	C17—C16—H16	119.8
C1—C6—C5	118.58 (19)	C15—C16—H16	119.8
C1—C6—C7	112.68 (17)	O2—C10—H10A	109.5
C5—C6—C7	128.74 (19)	O2—C10—H10B	109.5
C9—O2—C10	116.3 (2)	H10A—C10—H10B	109.5
C15—C14—C13	120.99 (18)	O2—C10—H10C	109.5
C15—C14—H14	119.5	H10A—C10—H10C	109.5
C13—C14—H14	119.5	H10B—C10—H10C	109.5
			
C13—C12—C21—C20	0.8 (3)	C11—C7—C6—C1	178.39 (16)
C11—C12—C21—C20	179.42 (16)	C8—C7—C6—C5	178.07 (19)
C17—C18—C13—C14	−1.5 (3)	C11—C7—C6—C5	−2.4 (3)
C19—C18—C13—C14	−179.66 (16)	C18—C13—C14—C15	0.3 (3)
C17—C18—C13—C12	177.65 (17)	C12—C13—C14—C15	−178.87 (19)
C19—C18—C13—C12	−0.5 (2)	C20—C19—C22—C23	1.1 (3)
C21—C12—C13—C14	178.28 (17)	C18—C19—C22—C23	−176.02 (19)
C11—C12—C13—C14	−0.4 (3)	C13—C18—C17—C16	1.7 (3)
C21—C12—C13—C18	−0.9 (2)	C19—C18—C17—C16	179.7 (2)
C11—C12—C13—C18	−179.58 (16)	C5—C6—C1—C2	0.7 (3)
C12—C21—C20—C19	0.8 (3)	C7—C6—C1—C2	179.98 (17)
C12—C21—C20—S2	−177.49 (14)	C5—C6—C1—S1	−179.07 (15)
C23—S2—C20—C19	0.66 (14)	C7—C6—C1—S1	0.2 (2)
C23—S2—C20—C21	179.08 (17)	C8—S1—C1—C2	−179.21 (19)
C21—C12—C11—C7	7.8 (3)	C8—S1—C1—C6	0.54 (15)
C13—C12—C11—C7	−173.54 (16)	C13—C14—C15—C16	0.9 (4)
C21—C20—C19—C22	−179.62 (16)	C10—O2—C9—O1	4.1 (4)
S2—C20—C19—C22	−1.1 (2)	C10—O2—C9—C8	−175.28 (19)
C21—C20—C19—C18	−2.1 (3)	C7—C8—C9—O1	−2.9 (4)
S2—C20—C19—C18	176.38 (13)	S1—C8—C9—O1	179.0 (2)
C17—C18—C19—C20	−176.14 (18)	C7—C8—C9—O2	176.42 (19)
C13—C18—C19—C20	1.9 (2)	S1—C8—C9—O2	−1.6 (2)
C17—C18—C19—C22	0.9 (3)	C19—C22—C23—S2	−0.6 (2)
C13—C18—C19—C22	178.91 (18)	C20—S2—C23—C22	0.00 (18)
C1—S1—C8—C7	−1.23 (15)	C1—C6—C5—C4	−0.3 (3)
C1—S1—C8—C9	177.06 (16)	C7—C6—C5—C4	−179.4 (2)
C9—C8—C7—C6	−176.57 (18)	C4—C3—C2—C1	0.3 (4)
S1—C8—C7—C6	1.5 (2)	C6—C1—C2—C3	−0.7 (3)
C9—C8—C7—C11	3.9 (3)	S1—C1—C2—C3	179.00 (18)
S1—C8—C7—C11	−177.94 (14)	C6—C5—C4—C3	−0.1 (4)
C12—C11—C7—C8	−92.0 (2)	C2—C3—C4—C5	0.1 (4)
C12—C11—C7—C6	88.6 (2)	C18—C17—C16—C15	−0.5 (4)
C8—C7—C6—C1	−1.1 (2)	C14—C15—C16—C17	−0.8 (4)

### Methyl 3-[(naphtho[2,1-b]thiophen-5-yl)methyl]-1-benzothiophene-2-carboxylate Hydrogen-bond geometry (Å, º)

*Cg*2 and *Cg*4 are the centroids of the S2/C19/C20/C22/C23 and C12/C13/C18-C21 rings, respectively.

**Table:**

*D*—H···*A*	*D*—H	H···*A*	*D*···*A*	*D*—H···*A*
C11—H11*B*···O1	0.97	2.44	3.007 (3)	117
C10—H10*A*···*Cg*2^i^	0.96	2.86	3.397 (3)	116
C17—H17···*Cg*4^ii^	0.93	2.98	3.689 (2)	134

Symmetry codes: (i) −*x*+1, −*y*, −*z*+2; (ii) *x*, −*y*−1/2, *z*−1/2.
